# 6,7,8,9-Tetra­hydro-4b,9b-dihy­droxy­indano[1,2-*b*]indoline-9,10-dione monohydrate

**DOI:** 10.1107/S1600536810025328

**Published:** 2010-07-03

**Authors:** Muhammad Yaqub, Khalid Mahmood, M. Nawaz Tahir, Zahid Shafiq, Abdul Rauf

**Affiliations:** a Department of Chemistry, Bahauddin Zakariya University, Multan 60800, Pakistan; b Department of Physics, University of Sargodha, Sargodha, Pakistan; c The Islamia University of Bahawalpur, Department of Chemistry, Bahawalpur, Pakistan

## Abstract

In the title compound, C_15_H_13_NO_4_·H_2_O, the organic mol­ecule adopts a V-shaped conformation in which the dihedral angle between the five-membered rings is 68.33 (5)°. The cyclo­hexenone ring adopts an envelope conformation, with one of the methyl­ene C atoms displaced by 0.607 (4) Å from the plane through the other atoms. In the crystal, inter­molecular N—H⋯(O,O) and O—H⋯O hydrogen bonds link the components into (001) sheeets and C–H⋯O inter­actions and aromatic π–π stacking [centroid–centroid separation = 3.6176 (19) Å] help to consolidate the packing.

## Related literature

For background to ninhydrin, see: Friedman (1967 ▶); Moubasher (1948 ▶). For a related structure, see: Black *et al.* (1994 ▶).
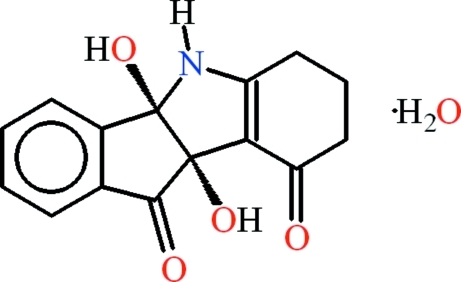


## Experimental

### 

#### Crystal data

C_15_H_13_NO_4_·H_2_O
*M* *_r_* = 289.28
Orthorhombic, 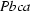

*a* = 10.703 (2) Å
*b* = 13.275 (4) Å
*c* = 19.683 (5) Å
*V* = 2796.6 (12) Å^3^
*Z* = 8
Mo *K*α radiation
μ = 0.10 mm^−1^
*T* = 296 K
0.30 × 0.22 × 0.18 mm

#### Data collection

Bruker Kappa APEXII CCD diffractometer
Absorption correction: multi-scan (*SADABS*; Bruker, 2005 ▶) *T*_min_ = 0.970, *T*_max_ = 0.978
17463 measured reflections
2532 independent reflections
1576 reflections with *I* > 2σ(*I*)
*R*_int_ = 0.066

#### Refinement

*R*[*F* ^2^ > 2σ(*F* ^2^)] = 0.047
*wR*(*F* ^2^) = 0.135
*S* = 1.08
2532 reflections
206 parameters
2 restraints
H atoms treated by a mixture of independent and constrained refinement
Δρ_max_ = 0.20 e Å^−3^
Δρ_min_ = −0.20 e Å^−3^

### 

Data collection: *APEX2* (Bruker, 2009 ▶); cell refinement: *SAINT* (Bruker, 2009 ▶); data reduction: *SAINT*; program(s) used to solve structure: *SHELXS97* (Sheldrick, 2008 ▶); program(s) used to refine structure: *SHELXL97* (Sheldrick, 2008 ▶); molecular graphics: *ORTEP-3 for Windows* (Farrugia, 1997 ▶) and *PLATON* (Spek, 2009 ▶); software used to prepare material for publication: *WinGX* (Farrugia, 1999 ▶) and *PLATON*.

## Supplementary Material

Crystal structure: contains datablocks global, I. DOI: 10.1107/S1600536810025328/hb5521sup1.cif

Structure factors: contains datablocks I. DOI: 10.1107/S1600536810025328/hb5521Isup2.hkl

Additional supplementary materials:  crystallographic information; 3D view; checkCIF report

## Figures and Tables

**Table 1:** Hydrogen-bond geometry (Å, °)

*D*—H⋯*A*	*D*—H	H⋯*A*	*D*⋯*A*	*D*—H⋯*A*
N1—H1⋯O1^i^	0.88 (3)	2.09 (3)	2.887 (3)	150 (2)
N1—H1⋯O3^i^	0.88 (3)	2.55 (3)	3.159 (3)	127 (2)
O2—H2*A*⋯O5^ii^	0.87 (3)	1.86 (3)	2.720 (3)	168 (3)
O4—H4*A*⋯O2^iii^	0.84 (3)	1.88 (3)	2.712 (3)	171 (3)
O5—H51⋯O3^iv^	0.94 (3)	1.83 (3)	2.762 (4)	174 (3)
C2—H2⋯O1^i^	0.93	2.46	3.052 (3)	122
C4—H4⋯O4^v^	0.93	2.34	3.253 (4)	165
C13—H13*A*⋯O3^i^	0.97	2.39	3.265 (4)	149

Symmetry codes: (i) 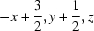; (ii) 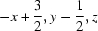; (iii) 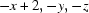; (iv) 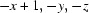; (v) 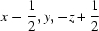.

**Fig. 1.**
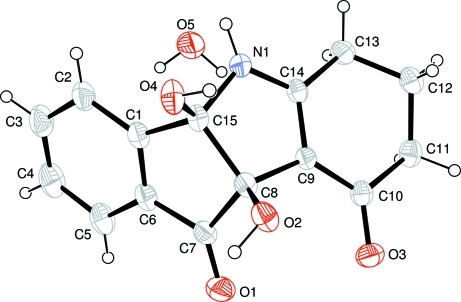
View of (I) with displacement ellipsoids drawn at the 30% probability level. H-atoms are shown by circles of arbitrary radius.

**Fig. 2.**
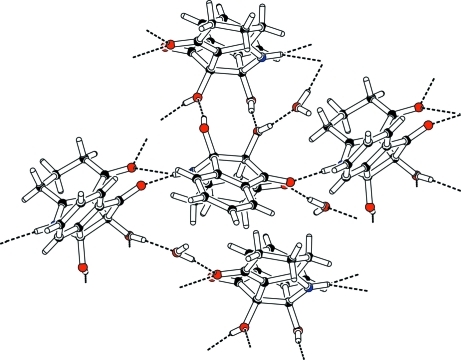
The partial packing of (I).

## supplementary crystallographic information

### Comment

The reaction of ninhydrin with 4-aminophenol in acetic acid, or 4-amino benzoic acid in benzene gave the corresponding 2-hydroxy-2-anilino-indane-1,3-diones (Moubasher *et al.*, 1948). Friedman (1967) elaborated on these findings and reported that *ortho* and *para* activated anilines gave imines corresponding to the dehydration products of hydroxy compounds. Ninhydrin is used to detect α-amino acids, proteins and dipeptides. The title compound (I), (Fig. 1) is being reported in connection with our plan to synthesize various derivatives of ninhydrin.

The crystal structure of (II) *i.e.* 5, 10-dihydro-7, 9-dimethoxy-4 b, 9 b, 10-trihydroxy-indeno[1,2-*b*]indole has been published (Black *et al.*, 1994). The compound (I) differs from (II) due to presence of two oxo groups instead of hydroxy and methoxy at position-9 & 10 respectively, H-atom instead of methoxy function at position-7 and due to presence of three hydrogen at position-6,7 & 8 of indole moiety.

In the organic part of title compound, there are two five membered and two six memberede rings. The carbon containing five membered A (C1/C6/C7/C8/C15) is fused with phenyl B (C1—C6) ring and with heterocyclic ring C (C15/C8/C9/C14/N1). The cyclohexenone ring D (C9—C14) is fused with the ring C. The ring A and B are planar with r. m. s. deviation of 0.0256 and 0.0091 Å, respectively and oriented at a dihedral angle of 3.07 (18)° with each other. The heterocyclic ring C is planar with r. m. s. deviation of 0.0163 Å. The group E (C9—C11/C13/C14) of cyclohexenone ring is also planar with r. m. s. deviation of 0.0206 Å and inclined with C at a dihedral angle of 1.55 (17)°. The C-atom labeled as C12 is at a distance of 0.6073 (40) Å from the mean square plane of E. There exist π···π interaction between rings B & C at a distance of 3.6176 (19)Å as the organic part is mainly in V-shape. The compound is stabilized due to complex form of H-bondings (Table 1, Fig. 2).

### Experimental

3-Amino-2-cyclohexene-1-one (0.10 g, 0.89 mmol) was added to a stirred solution of ninhydrin (0.16 g, 0.89 mmol) in propanol (10 ml) and heated under reflux for 35 minuts. After completion of reaction, the mixture was cooled at room temperature. The crystalline solid was collected by suction filtration. Through washing with hot ethanol afforded the white crystalline solid (0.22 g, 85%), m.p. 526 K.  Colourless prisms of (I) were grown by diffusion method in ethyl acetate:benzene (1:1) system along with few drops of ethanol.

### Refinement

The coordinates of H-atoms of amine and hydroxy groups were refined and the other H-atoms were positioned geometrically (C–H = 0.93–0.97 Å) and refined as riding with *U*_iso_(H) = *xU*_eq_(C, N, O), where *x* = 1.2 for all H-atoms.

### Crystal data

**Table:**

C_15_H_13_NO_4_·H_2_O	*F*(000) = 1216
*M**_r_* = 289.28	*D*_x_ = 1.369 Mg m^−^^3^
Orthorhombic, *P**b**c**a*	Mo *K*α radiation, λ = 0.71073 Å
Hall symbol: -P 2ac 2ab	Cell parameters from 1576 reflections
*a* = 10.703 (2) Å	θ = 2.7–25.3°
*b* = 13.275 (4) Å	µ = 0.10 mm^−^^1^
*c* = 19.683 (5) Å	*T* = 296 K
*V* = 2796.6 (12) Å^3^	Prism, colourless
*Z* = 8	0.30 × 0.22 × 0.18 mm

### Data collection

**Table:**

Bruker Kappa APEXII CCD diffractometer	2532 independent reflections
Radiation source: fine-focus sealed tube	1576 reflections with *I* > 2σ(*I*)
graphite	*R*_int_ = 0.066
Detector resolution: 8.20 pixels mm^-1^	θ_max_ = 25.5°, θ_min_ = 2.7°
ω scans	*h* = −12→12
Absorption correction: multi-scan (*SADABS*; Bruker, 2005)	*k* = −15→15
*T*_min_ = 0.970, *T*_max_ = 0.978	*l* = −23→23
17463 measured reflections	

### Refinement

**Table:**

Refinement on *F*^2^	Primary atom site location: structure-invariant direct methods
Least-squares matrix: full	Secondary atom site location: difference Fourier map
*R*[*F*^2^ > 2σ(*F*^2^)] = 0.047	Hydrogen site location: inferred from neighbouring sites
*wR*(*F*^2^) = 0.135	H atoms treated by a mixture of independent and constrained refinement
*S* = 1.08	*w* = 1/[σ^2^(*F*_o_^2^) + (0.0498*P*)^2^ + 1.2061*P*] where *P* = (*F*_o_^2^ + 2*F*_c_^2^)/3
2532 reflections	(Δ/σ)_max_ = 0.001
206 parameters	Δρ_max_ = 0.20 e Å^−^^3^
2 restraints	Δρ_min_ = −0.20 e Å^−^^3^

### Special details

**Table:**

Geometry. Bond distances, angles *etc*. have been calculated using the rounded fractional coordinates. All su's are estimated from the variances of the (full) variance-covariance matrix. The cell e.s.d.'s are taken into account in the estimation of distances, angles and torsion angles
Refinement. Refinement of *F*^2^ against ALL reflections. The weighted *R*-factor *wR* and goodness of fit *S* are based on *F*^2^, conventional *R*-factors *R* are based on *F*, with *F* set to zero for negative *F*^2^. The threshold expression of *F*^2^ > σ(*F*^2^) is used only for calculating *R*-factors(gt) *etc*. and is not relevant to the choice of reflections for refinement. *R*-factors based on *F*^2^ are statistically about twice as large as those based on *F*, and *R*- factors based on ALL data will be even larger.

### Fractional atomic coordinates and isotropic or equivalent isotropic displacement parameters (Å^2^)

**Table:**

	*x*	*y*	*z*	*U*_iso_*/*U*_eq_	
O1	0.6918 (2)	−0.15555 (15)	0.11236 (10)	0.0602 (8)	
O2	0.91068 (17)	−0.08706 (14)	0.03362 (9)	0.0464 (7)	
O3	0.64800 (18)	−0.14565 (14)	−0.04188 (10)	0.0540 (7)	
O4	0.98666 (17)	0.09354 (16)	0.09227 (9)	0.0498 (7)	
N1	0.8012 (2)	0.15460 (17)	0.04090 (11)	0.0402 (7)	
C1	0.7997 (2)	0.0847 (2)	0.15639 (13)	0.0416 (9)	
C2	0.8110 (3)	0.1600 (2)	0.20487 (14)	0.0563 (11)	
C3	0.7442 (4)	0.1498 (3)	0.26471 (15)	0.0681 (13)	
C4	0.6683 (3)	0.0675 (3)	0.27637 (16)	0.0692 (13)	
C5	0.6593 (3)	−0.0089 (3)	0.22942 (14)	0.0582 (11)	
C6	0.7266 (2)	0.0012 (2)	0.16905 (12)	0.0430 (9)	
C7	0.7359 (2)	−0.07177 (19)	0.11328 (13)	0.0400 (9)	
C8	0.8128 (2)	−0.02380 (18)	0.05584 (12)	0.0366 (8)	
C9	0.7294 (2)	0.00822 (18)	−0.00174 (12)	0.0330 (8)	
C10	0.6574 (2)	−0.0522 (2)	−0.04613 (13)	0.0395 (9)	
C11	0.5833 (3)	0.0030 (2)	−0.10052 (14)	0.0482 (10)	
C12	0.6310 (3)	0.1070 (2)	−0.11767 (14)	0.0518 (10)	
C13	0.6515 (3)	0.16996 (19)	−0.05470 (13)	0.0450 (9)	
C14	0.7286 (2)	0.11153 (18)	−0.00559 (12)	0.0353 (8)	
C15	0.8580 (2)	0.08050 (19)	0.08678 (12)	0.0382 (8)	
O5	0.4998 (3)	0.2705 (2)	0.12020 (14)	0.0943 (11)	
H1	0.808 (2)	0.220 (2)	0.0473 (13)	0.0483*	
H2	0.86192	0.21568	0.19739	0.0676*	
H2A	0.932 (3)	−0.129 (2)	0.0658 (15)	0.0556*	
H3	0.75062	0.19956	0.29781	0.0817*	
H4	0.62257	0.06369	0.31648	0.0830*	
H4A	1.018 (3)	0.085 (2)	0.0537 (15)	0.0597*	
H5	0.61011	−0.06532	0.23762	0.0701*	
H11A	0.58359	−0.03738	−0.14158	0.0578*	
H11B	0.49724	0.00876	−0.08548	0.0578*	
H12A	0.57126	0.14056	−0.14700	0.0621*	
H12B	0.70914	0.10108	−0.14231	0.0621*	
H13A	0.69391	0.23208	−0.06659	0.0540*	
H13B	0.57173	0.18695	−0.03431	0.0540*	
H51	0.452 (3)	0.224 (2)	0.0954 (17)	0.1131*	
H52	0.526 (4)	0.239 (3)	0.1564 (14)	0.1131*	

### Atomic displacement parameters (Å^2^)

**Table:**

	*U*^11^	*U*^22^	*U*^33^	*U*^12^	*U*^13^	*U*^23^
O1	0.0834 (15)	0.0458 (13)	0.0515 (13)	−0.0033 (11)	0.0135 (11)	0.0065 (10)
O2	0.0484 (11)	0.0525 (12)	0.0382 (11)	0.0207 (9)	0.0053 (9)	0.0057 (9)
O3	0.0633 (13)	0.0389 (11)	0.0598 (13)	−0.0041 (9)	−0.0089 (10)	−0.0009 (10)
O4	0.0396 (11)	0.0760 (14)	0.0337 (10)	−0.0043 (9)	−0.0021 (8)	−0.0020 (10)
N1	0.0501 (13)	0.0359 (12)	0.0347 (12)	−0.0002 (10)	−0.0058 (10)	0.0002 (10)
C1	0.0455 (15)	0.0480 (17)	0.0314 (14)	0.0096 (13)	−0.0008 (12)	0.0009 (12)
C2	0.077 (2)	0.0570 (19)	0.0349 (16)	0.0064 (16)	−0.0027 (15)	−0.0028 (14)
C3	0.099 (3)	0.068 (2)	0.0373 (18)	0.020 (2)	−0.0008 (18)	−0.0101 (16)
C4	0.073 (2)	0.097 (3)	0.0377 (17)	0.015 (2)	0.0149 (16)	0.0000 (18)
C5	0.0555 (19)	0.080 (2)	0.0392 (16)	0.0044 (16)	0.0089 (14)	0.0058 (16)
C6	0.0424 (16)	0.0544 (17)	0.0321 (14)	0.0108 (13)	0.0018 (12)	0.0031 (13)
C7	0.0449 (15)	0.0399 (16)	0.0352 (15)	0.0082 (12)	0.0020 (12)	0.0054 (12)
C8	0.0376 (14)	0.0393 (14)	0.0328 (14)	0.0089 (11)	0.0035 (11)	0.0011 (11)
C9	0.0342 (13)	0.0357 (14)	0.0291 (12)	0.0029 (11)	0.0013 (10)	0.0014 (10)
C10	0.0373 (15)	0.0454 (16)	0.0357 (14)	0.0021 (12)	0.0034 (12)	−0.0018 (12)
C11	0.0462 (16)	0.0570 (18)	0.0414 (16)	0.0024 (14)	−0.0084 (13)	−0.0042 (14)
C12	0.0590 (18)	0.0549 (18)	0.0415 (16)	0.0024 (14)	−0.0110 (14)	0.0083 (14)
C13	0.0486 (16)	0.0420 (16)	0.0445 (16)	0.0051 (12)	−0.0083 (13)	0.0063 (12)
C14	0.0347 (14)	0.0417 (15)	0.0296 (13)	0.0015 (11)	0.0030 (11)	0.0009 (11)
C15	0.0385 (15)	0.0460 (16)	0.0301 (13)	0.0030 (12)	−0.0009 (11)	0.0001 (11)
O5	0.122 (2)	0.090 (2)	0.0708 (18)	−0.0540 (17)	−0.0238 (16)	0.0200 (14)

### Geometric parameters (Å, °)

**Table:**

O1—C7	1.208 (3)	C7—C8	1.537 (3)
O2—C8	1.412 (3)	C8—C9	1.504 (3)
O3—C10	1.247 (3)	C8—C15	1.588 (3)
O4—C15	1.392 (3)	C9—C14	1.374 (3)
O2—H2A	0.87 (3)	C9—C10	1.414 (3)
O4—H4A	0.84 (3)	C10—C11	1.521 (4)
O5—H51	0.94 (3)	C11—C12	1.510 (4)
O5—H52	0.87 (3)	C12—C13	1.511 (4)
N1—C14	1.330 (3)	C13—C14	1.489 (4)
N1—C15	1.467 (3)	C2—H2	0.9300
N1—H1	0.88 (3)	C3—H3	0.9300
C1—C6	1.380 (4)	C4—H4	0.9300
C1—C2	1.387 (4)	C5—H5	0.9300
C1—C15	1.507 (3)	C11—H11B	0.9700
C2—C3	1.385 (4)	C11—H11A	0.9700
C3—C4	1.381 (6)	C12—H12A	0.9700
C4—C5	1.376 (5)	C12—H12B	0.9700
C5—C6	1.396 (4)	C13—H13B	0.9700
C6—C7	1.467 (4)	C13—H13A	0.9700
			
C8—O2—H2A	110 (2)	C12—C13—C14	109.0 (2)
C15—O4—H4A	108 (2)	N1—C14—C13	123.1 (2)
H51—O5—H52	107 (3)	N1—C14—C9	112.8 (2)
C14—N1—C15	112.2 (2)	C9—C14—C13	124.0 (2)
C15—N1—H1	122.6 (16)	N1—C15—C8	102.84 (18)
C14—N1—H1	124.8 (16)	C1—C15—C8	104.77 (19)
C2—C1—C6	120.3 (2)	O4—C15—N1	112.0 (2)
C2—C1—C15	128.0 (2)	O4—C15—C1	109.54 (19)
C6—C1—C15	111.7 (2)	N1—C15—C1	111.31 (19)
C1—C2—C3	118.0 (3)	O4—C15—C8	116.07 (19)
C2—C3—C4	121.5 (3)	C1—C2—H2	121.00
C3—C4—C5	120.9 (3)	C3—C2—H2	121.00
C4—C5—C6	117.7 (3)	C4—C3—H3	119.00
C5—C6—C7	127.5 (3)	C2—C3—H3	119.00
C1—C6—C5	121.6 (3)	C3—C4—H4	120.00
C1—C6—C7	110.9 (2)	C5—C4—H4	120.00
C6—C7—C8	108.3 (2)	C4—C5—H5	121.00
O1—C7—C6	126.3 (2)	C6—C5—H5	121.00
O1—C7—C8	125.4 (2)	C10—C11—H11B	109.00
O2—C8—C9	112.03 (19)	C12—C11—H11A	109.00
O2—C8—C7	112.26 (19)	C12—C11—H11B	108.00
C7—C8—C9	110.71 (18)	H11A—C11—H11B	108.00
C7—C8—C15	104.02 (19)	C10—C11—H11A	109.00
C9—C8—C15	102.91 (19)	C11—C12—H12B	109.00
O2—C8—C15	114.29 (18)	C13—C12—H12A	109.00
C8—C9—C14	109.1 (2)	C11—C12—H12A	109.00
C10—C9—C14	121.9 (2)	H12A—C12—H12B	108.00
C8—C9—C10	129.0 (2)	C13—C12—H12B	109.00
O3—C10—C9	124.5 (2)	C12—C13—H13A	110.00
O3—C10—C11	119.0 (2)	C12—C13—H13B	110.00
C9—C10—C11	116.5 (2)	C14—C13—H13B	110.00
C10—C11—C12	114.9 (2)	H13A—C13—H13B	108.00
C11—C12—C13	111.8 (2)	C14—C13—H13A	110.00
			
C15—N1—C14—C9	−3.2 (3)	C6—C7—C8—C15	5.2 (2)
C15—N1—C14—C13	174.7 (2)	O2—C8—C9—C10	60.0 (3)
C14—N1—C15—O4	129.3 (2)	O2—C8—C9—C14	−121.6 (2)
C14—N1—C15—C1	−107.7 (2)	C7—C8—C9—C10	−66.1 (3)
C14—N1—C15—C8	4.0 (2)	C7—C8—C9—C14	112.3 (2)
C6—C1—C2—C3	−1.9 (4)	C15—C8—C9—C10	−176.7 (2)
C15—C1—C2—C3	177.3 (3)	C15—C8—C9—C14	1.7 (2)
C2—C1—C6—C5	2.0 (4)	O2—C8—C15—O4	−4.1 (3)
C2—C1—C6—C7	−175.7 (2)	O2—C8—C15—N1	118.5 (2)
C15—C1—C6—C5	−177.3 (2)	O2—C8—C15—C1	−125.1 (2)
C15—C1—C6—C7	5.1 (3)	C7—C8—C15—O4	118.6 (2)
C2—C1—C15—O4	54.1 (3)	C7—C8—C15—N1	−118.75 (19)
C2—C1—C15—N1	−70.3 (3)	C7—C8—C15—C1	−2.3 (2)
C2—C1—C15—C8	179.3 (2)	C9—C8—C15—O4	−125.8 (2)
C6—C1—C15—O4	−126.7 (2)	C9—C8—C15—N1	−3.2 (2)
C6—C1—C15—N1	108.9 (2)	C9—C8—C15—C1	113.25 (19)
C6—C1—C15—C8	−1.5 (3)	C8—C9—C10—O3	3.4 (4)
C1—C2—C3—C4	0.0 (5)	C8—C9—C10—C11	−179.2 (2)
C2—C3—C4—C5	1.9 (6)	C14—C9—C10—O3	−174.8 (2)
C3—C4—C5—C6	−1.8 (5)	C14—C9—C10—C11	2.6 (3)
C4—C5—C6—C1	−0.1 (4)	C8—C9—C14—N1	0.8 (3)
C4—C5—C6—C7	177.1 (3)	C8—C9—C14—C13	−177.1 (2)
C1—C6—C7—O1	173.0 (2)	C10—C9—C14—N1	179.3 (2)
C1—C6—C7—C8	−6.5 (3)	C10—C9—C14—C13	1.4 (4)
C5—C6—C7—O1	−4.4 (4)	O3—C10—C11—C12	−160.4 (2)
C5—C6—C7—C8	176.1 (3)	C9—C10—C11—C12	22.0 (3)
O1—C7—C8—O2	−50.3 (3)	C10—C11—C12—C13	−49.8 (3)
O1—C7—C8—C9	75.7 (3)	C11—C12—C13—C14	50.8 (3)
O1—C7—C8—C15	−174.4 (2)	C12—C13—C14—N1	153.7 (2)
C6—C7—C8—O2	129.2 (2)	C12—C13—C14—C9	−28.7 (3)
C6—C7—C8—C9	−104.8 (2)		

### Hydrogen-bond geometry (Å, °)

**Table:**

*D*—H···*A*	*D*—H	H···*A*	*D*···*A*	*D*—H···*A*
N1—H1···O1^i^	0.88 (3)	2.09 (3)	2.887 (3)	150 (2)
N1—H1···O3^i^	0.88 (3)	2.55 (3)	3.159 (3)	127 (2)
O2—H2A···O5^ii^	0.87 (3)	1.86 (3)	2.720 (3)	168 (3)
O4—H4A···O2^iii^	0.84 (3)	1.88 (3)	2.712 (3)	171 (3)
O5—H51···O3^iv^	0.94 (3)	1.83 (3)	2.762 (4)	174 (3)
C2—H2···O1^i^	0.93	2.46	3.052 (3)	122
C4—H4···O4^v^	0.93	2.34	3.253 (4)	165
C13—H13A···O3^i^	0.97	2.39	3.265 (4)	149

Symmetry codes: (i) −*x*+3/2, *y*+1/2, *z*; (ii) −*x*+3/2, *y*−1/2, *z*; (iii) −*x*+2, −*y*, −*z*; (iv) −*x*+1, −*y*, −*z*; (v) *x*−1/2, *y*, −*z*+1/2.
